# 3D-Printed Ginsenoside Rb1-Loaded Mesoporous Calcium Silicate/Calcium Sulfate Scaffolds for Inflammation Inhibition and Bone Regeneration

**DOI:** 10.3390/biomedicines9080907

**Published:** 2021-07-28

**Authors:** Cheng-Yu Chen, Ming-You Shie, Alvin Kai-Xing Lee, Yun-Ting Chou, Chun Chiang, Chun-Pin Lin

**Affiliations:** 1 Graduate Institute of Clinical Dentistry, School of Dentistry, National Taiwan University, Taipei 10617, Taiwan; k55599911@gmail.com; 2 School of Dentistry, China Medical University, Taichung City 406040, Taiwan; eric@mail.cmu.edu.tw (M.-Y.S.); u109000407@cmu.edu.tw (C.C.); 3 x-Dimension Center for Medical Research and Translation, China Medical University Hospital, Taichung 404332, Taiwan; Leekaixingalvin@gmail.com; 4 Department of Bioinformatics and Medical Engineering, Asia University, Taichung City 41354, Taiwan; 5 School of Medicine, China Medical University, Taichung 406040, Taiwan; 6 Graduate Institute of Dental Science and Oral Health Industries, China Medical University, Taichung 406040, Taiwan; tingsmile666@gmail.com; 7 Department of Dentistry, National Taiwan University Hospital, Taipei 100229, Taiwan; 8 School of Dentistry, College of Dental Medicine, Kaohsiung Medical University, Kaohsiung 807378, Taiwan

**Keywords:** ginsenoside Rb1, mesoporous calcium silicate, calcium sulfate, 3D printing, inflammation

## Abstract

Bone defects are commonly found in the elderly and athletic population due to systemic diseases such as osteoporosis and trauma. Bone scaffolds have since been developed to enhance bone regeneration by acting as a biological extracellular scaffold for cells. The main advantage of a bone scaffold lies in its ability to provide various degrees of structural support and growth factors for cellular activities. Therefore, we designed a 3D porous scaffold that can not only provide sufficient mechanical properties but also carry drugs and promote cell viability. Ginsenoside Rb1 (GR) is an extract from panax ginseng, which has been used for bone regeneration and repair since ancient Chinese history. In this study, we fabricated scaffolds using various concentrations of GR with mesoporous calcium silicate/calcium sulfate (MSCS) and investigated the scaffold’s physical and chemical characteristic properties. PrestoBlue, F-actin staining, and ELISA were used to demonstrate the effect of the GR-contained MSCS scaffold on cell proliferation, morphology, and expression of the specific osteogenic-related protein of human dental pulp stem cells (hDPSCs). According to our data, hDPSCs cultivated in GR-contained MSCS scaffold had preferable abilities of proliferation and higher expression of the osteogenic-related protein and could effectively inhibit inflammation. Finally, in vivo performance was assessed using histological results that revealed the GR-contained MSCS scaffolds were able to further achieve more effective hard tissue regeneration than has been the case in the past. Taken together, this study demonstrated that a GR-containing MSCS 3D scaffold could be used as a potential alternative for future bone tissue engineering studies and has good potential for clinical use.

## 1. Introduction

Bone diseases are the main cause of disability and are commonly found amongst the elderly. According to 2017–2018 osteoporosis data from the Centers for Disease Control and Prevention (America), 4.2% of men and 18.8% of women aged 50 years and above had a systemic bone loss of the femur neck or lumbar spine. A common complication for systemic bone diseases is fracture due to weakened bone integrity and structural loss. The current treatment method for such bone defects includes bone autografts and allografts [1]. However, these treatment methods have severe limitations restricting their fullest potential in treating bone defects [2]. The elderly with systemic bone diseases often have limited suitable grafting locations, and they usually have chronic diseases, which makes them unsuitable for additional harvesting or prolonged surgical procedures [3]. In order to overcome systemic side effects of medications and at the same time be able to stimulate bone regeneration, scientists have attempted to develop a stable drug-carrier system for the local release of osteoinduction and osteogenic factors [4]. To date, many reports regarding drug carrier systems have been published, and various types of material and scaffolds have been applied to investigate the effects of biocompatibility and osteogenic potential between bone tissue and bone defect site [5,6,7].

In addition, three-dimensional (3D) printing has gained popularity in this field of study due to its capability to personalize and customize scaffolds for large bone defects and wound repair [8,9,10]. Furthermore, interconnected nanopores could be fabricated using 3D printing, with nanopores reported as being able to provide a suitable microenvironment for cell proliferation and attachment [11]. Multiple types of biomaterials such as hydrogel, ceramic, and metal have since been explored for clinical bone regeneration and repair [12,13,14]. Calcium silicate-based ceramic (CS) has demonstrated its expressed biocompatibility and osteoinductivity compared with calcium phosphate bioceramic, which can urge osteogenic differentiation of pre-osteoblasts by promoting the secretion of functional proteins [15,16]. Among them, the main reason is the presence of the silicon ion (Si), which has been found to modulate the activity of cellular behaviors, such as proliferation, differentiation, and mineralization of primary cells [17,18]. In addition, Si can also support adhesion interactions between cells and scaffolds, as well as promote angiogenesis-related functions of human umbilical vein endothelial cells [19]. Our previous studies have shown that mesoporous calcium silicate/calcium sulfate scaffold (MSCS) could provide a favorable microenvironment for cell proliferation, differentiation, and bone mineralization [20]. In addition, MSCS scaffolds showed anti-bacterial effects and had enhanced mechanical properties, which were able to support long-term drug release and enhanced bone regeneration in animal studies. It was further hypothesized that the effects of MSCS were due to the pores and capability to release ions such as calcium and silicate ions. Furthermore, we attempted to load BMP-2 onto MSCS, and our results showed that MSCS is an excellent and stable candidate for drug loading and release to enhance bioactivity [21].

Traditional and natural medicines have been around for thousands of years and several medicinal compounds were effective in treating certain types of diseases [22]. Ginsenoside (GR) is a traditional stem extract that was found to have various physiological and biological functions, such as anti-oxidative, anti-inflammatory, and angiogenic characteristics [23,24]. GR can be classified into several subgroups, of which Rb1, Rg1, and Re are believed to have effects on increasing activities of osteoblasts and osteoclasts, thus leading to enhanced bone tissue healing [25]. GR is thus commonly used in clinical settings for osteoporotic patients. In addition, a high dosage of GR was found to induce BMP-2/SMAD signaling, which further enhanced bone mineral density, bone volume, and bone trabecular thickness through activation of rat bone marrow mesenchymal stem cell osteogenic differentiation [26]. Additionally, GR was proven to play a role in regulating proliferation, differentiation, and apoptosis of in vitro and in vivo mesenchymal stem cells [27]. Moreover, GR showed excellent angiogenesis and bone regeneration on dental implant models of rat mandibles [28]. However, according to our knowledge, reports were rare regarding the combination of GR and 3D-printed scaffolds in evaluating biocompatibility and treatment of bone defects. 

The aim of this study was to explore the drug release and bone regenerative capabilities of 3D-printed porous GR-contained scaffolds, which could improve stability and promote bone regeneration. The goal was to design novel bone substitute scaffolds and to assess for their physical and chemical characteristics, such as material morphology, composition, and mechanical properties. In addition, cell behaviors, such as the viability, proliferation, mineralization, and osteogenic-related markers, of human dental pulp stem cells (hDPSCs) cultured on the scaffold were investigated. Finally, we examined the potential of GR scaffolds in femur defects of animal models, which revealed that the GR bioscaffold was able to promote hDPSC’s cell-to-cell interaction and bone tissue regeneration and also revealed that the GR bioscaffold might be a high potential bone substitute scaffold in the clinical setting.

## 2. Materials and Methods

### 2.1. Preparation of the GR-Contained MSCS Powder

MSCS ceramic composites were fabricated according to our previous study [20]. Firstly, 3.3 g cetyltrimethylammonium bromide (CTAB, Sigma-Aldrich, St. Louis, MO, USA) was blended with 6 mL NH_3_·H_2_O in distilled water (ddH_2_O, 300 mL) and stirred for 15 min at 60 °C. Secondly, tetraethyl orthosilicate (Sigma-Aldrich, St. Louis, MO, USA) 15 mL and calcium nitrate 15.6 g were mixed, then stirred slowly until homogenized for 3 h. Next, ethanol and 1 N hydrochloric acid were used for product precipitation. The powders were dried at 60 °C in an oven overnight, then set to 800 °C for 2 h and sintered to remove the remaining CTAB. Subsequently, CS powder (Sigma-Aldrich, St. Louis, MO, USA), MS powder, and ginsenoside Rb1 (GR, Biomed, Taichung, Taiwan) were mixed and milled in a planetary ball (Retsch PM-100, Retsch GmbH, Haan, Germany) under a 99% ethanol solution for 8 h; then, raw ceramic composite powders were obtained via drying for 12 h.

### 2.2. GR-Contained MSCS Scaffolds Fabrication

The scaffold was fabricated by using fused deposition modeling (FDM) technology. Firstly, we put reagent grade polycaprolactone (PCL) (molecular weight = 43,000–50,000; Polysciences, Warrington, PA, USA) in the oven for one hour and set the temperature to 100 °C. The ratios of the GR/MSCS/PCL composite had divided into three groups, 0:50:50, 2:48:50, and 5:45:50; we named the codes as G0, G2, and G5, respectively. These composites were mixed in 99.5% alcohol and dripped gently into the melted PCL. The solution was then placed at 100 °C and stirred rapidly for 1 h to promote alcohol evaporation at room temperature. The 3D scaffold was fabricated by Bio-Scaffolder 3.1 (GeSiM, Grosserkmannsdorf, Radeberg, Germany) with a stable pressure of 330 kPa. All scaffolds were printed in parallel, with 400 μm struts, 300 μm height, and 500 μm spacing between each layer.

### 2.3. Physical and Chemical Properties of the Scaffolds

The scaffold surface phase structure was detected by X-ray diffractometer analysis (XRD, Shimadzu Corporation, Kyoto, Japan). The condition was as follows: set 1° for divergence and scatter slits with a receiving slot of 0.10 mm. The scan range was set from 20° to 50° and at θ to 2θ with a scan speed of 1°/min. In addition, functional group change was detected via Fourier-transform infrared spectrometer (FTIR, Bruker, Ettlingen, Germany), with the spectral range set between 500 cm^−1^ to 4000 cm^−1^ and a reflection absorption mode of 1 cm^−1^. Mechanical properties were assessed using an EZ Test machine (Shimadzu, Kyoto, Japan); the load-bearing condition of each scaffold was set to be tested at 1 mm/min until scaffold fracture occurred. The maximal compressive strength was evaluated based on the recorded stress-strain curves. All trials were performed in six repetitions and values were recorded. A field emission scanning electron microscope (SEM, JEOL, Tokyo, Japan) was used to observe the microstructure morphology of the scaffolds.

### 2.4. In Vitro Soaking

Next, the scaffold was immersed in simulated body fluid (SBF) to understand if beneficial crystals can be precipitated. The detailed composition of SBF is as follows; 7.9949 g NaCl, 0.2235 g KCl, 0.147 g K_2_HPO_4_, 0.3528 g NaHCO_3_, 0.071 g Na_2_SO_4_, 0.2775 g CaCl_2_, and 0.305 g MgCl_2_ ∙6H_2_O in 1000 mL of distilled H_2_O. Then, we used pH hydrochloric acid and tris(hydroxymethyl)aminomethane to control the pH, such that pH = 7.4. The scaffolds were soaked and taken out at different time points. The microstructure of the scaffolds’ surface was then investigated by scanning electron microscope. In addition, Si ion released into the medium was considered using an inductive coupled plasma-atomic emission spectrometer (ICP-AES; Perkin-Elmer OPT 1MA 3000DV, Shelton, CT, USA).

### 2.5. Cell Adhesion and Proliferation

Human dental pulp stem cells (hDPSCs) were purchased from Lonza (PT-5025, Lonza, Basel, Switzerland) in a commercially-available human dental pulp stem cell bullet kit (PT-3005, Lonza, Basel, Switzerland). New culture medium was replaced twice a day in a 37 °C humidified atmosphere with 5% CO_2_. The cells started the experiment when the culture reached passage 3–6; the 3D scaffolds were pretreated with 75% ethanol for 1h, then subjected to ultraviolet light exposure for 30 min before the cell study. After the various duration of cultures, the PrestoBlue assay (Invitrogen, Carlsbad, CA, USA) was utilized, following the manufacturer’s manual instructions to experiment. Internal mitochondrial activity was investigated according to the color intensity of the reagent. The viability value was measured by a multi-well spectrophotometer (Infinite Pro M200, Tecan, Männedorf, Switzerland) at 570 nm with a reference wavelength of 600 nm. The only hDPSCs seeding was the control group.

### 2.6. Fluorescent Staining

After pretreatment, we seeded and cultured the hDPSCs on the scaffold for 1 and 3 days; the specimens were washed by phosphate-buffered saline (PBS, Invitrogen, Carlsbad, CA, USA) three times, then fixed in 4% paraformaldehyde (Sigma-Aldrich, St. Louis, MO, USA) for 15 min, then exposed to 0.1% Triton X-100 (Sigma-Aldrich, St. Louis, MO, USA) for 15 min at room temperature. The cytoskeletal morphology was observed by F-actin fluoresce and was stained with phalloidin conjugated to Alexa Fluor 488 (Invitrogen, Carlsbad, CA, USA) for 1 h. DAPI (Invitrogen, Carlsbad, CA, USA) was stained with a nucleus for 30 min. During each step, PBS washes were performed three times. Finally, a white light laser confocal microscope (TCS SP8; Leica, Wetzlar, Germany) was used to determine cell morphology.

### 2.7. Enzyme-Linked Immunosorbent Assay

After hDPSCs were cultured on the scaffold, the cell was lysed by NP40 and we collected protein for considering the immune-related and osteogenic-related marker. In the osteogenic assay, the hDPSCs were cultured with osteogenesis assay kits (StemPro™ osteogenesis differentiation kit, Invitrogen, Carlsbad, CA, USA) were used for estimation of cell differentiation. The expression of alkaline phosphatase (ALP), osteopontin (OPN), and osteocalcin (OC) were induced by GR composite scaffolds on days 3 and 7 after the cell was cultured in osteogenic medium. Cells were added to 0.2% NP40 to cause lysis and were then centrifuged at 6000 rpm for 15 min. ALP activity was determined using p-nitrophenyl phosphate (Sigma-Aldrich, St. Louis, MO, USA) as the substrate. Each sample was mixed with p-nitrophenyl phosphate in 1 mol/L diethanolamine buffer. ALP expression was determined by p-nitrophenyl phosphate (Sigma-Aldrich, St. Louis, MO, USA). The steps were as followes: cells were treated with 0.2% NP40, centrifuged at 6000 rpm for 15 min, then blended in 1 mol/L diethanolamine buffer to each specimen, respectively. Finally, we added the 3M NaOH to terminate the reaction after 30 min. Results quantified by spectrophotometer with absorbance at 405 nm. The quantity of IL-1β, IL-1RA, OPN, and OC (MyBioSource, San Diego, CA, USA) secreted from the hDPSCs at different time points were determined using enzyme-linked immunosorbent assay according to the manufacturer’s instructions. The concentration of the standard curve was investigated through BCA assay, and the blank well was the control group.

### 2.8. Mineralization

The mineralization of hDPSCs was assayed by Alizarin Red S. The seeding cell on the scaffold was then cultured in commercial osteogenic differentiation medium for 7 and 14 days. Briefly, samples were treated with 4% paraformaldehyde (Sigma-Aldrich, St. Louis, MO, USA) for 10 min to fix the cell, then PBS washed three times to avoid interference and stained with 0.5% Alizarin Red S (pH = 4.0, Sigma-Aldrich, St. Louis, MO, USA) for 15 min. The mineralization images were detected by white light laser confocal microscope to obtain cell morphology.

### 2.9. Rabbit Model of Femoral Bone Defects

The in vivo experimental protocol was approved by the Animal Experimental Ethics Committee of China Medical University in Taichung, Taiwan (CMUIACUC-2019-099-1). Healthy New Zealand rabbits, weighing about 1.8–2 kg, were selected at the age of three months for the femoral defect assay. All rabbits were purchased from the National Animal Center of Taiwan (Taipei, Taiwan). The rabbits were divided into two groups of three rabbits each. The rabbits in one group were implanted with a scaffold containing GR at a concentration of 5%, while the other group did not have any GR. First, we injected chlorhexidine into the rabbits, then released 100% oxygen and 5% isoflurane by a gas anesthesia machine to stably anesthetize the animals. Before implantation, hair on the hind legs was removed, and alcohol and iodine was utilized to sterilize the skin surface. Second, the fascia was removed, and avoiding the nerves and blood vessels, a bone drill was utilized directly on the femur to create bone defects. The wound created by the implanted stent was to be sutured and the femoral area was to be taken out according to the time point to sacrifice the animal to observe the degree of bone repair at the implantation GR scaffold site. All rabbits were fasting for 1 day before the operation.

### 2.10. Histological Staining

The samples were retrieved following protocols approved by our ethical committee after implantation of the scaffolds at 4 and 8 weeks, respectively. The samples were cleaned, fixed, and finally sectioned at 6 mm apiece (OCT^®^) (KMA-0100-00A, CellPath Ltd., Newtown, Wales, UK). Then, microtome was used to prepare 6 μm sections from each sample. All section’s morphology integrity was then stained with hematoxylin and eosin (H&E, ScyTek Lab, West Logan, UT, USA), Masson’s trichrome stain kit (MT, ScyTek Lab, West Logan, UT, USA), and von Kossa kit (VK, ScyTek Lab, West Logan, UT, USA). The trichrome staining in blue was measured to identify collagen distribution. The Von Kossa was expressed dark brown which allowed the observation of the mineralization of osteoid tissue and peri-calcified bone. All sample staining was detected by microscope (BX53, Olympus, Tokyo, Japan).

### 2.11. Statistical Analyses

Significant differences were analyzed by a one-way variance statistical analysis (ANOVA) of at least three experiments. Statistical comparisons of more than two groups were performed investigating Scheffe’s multiple comparison test. In all cases, the *p*-value < 0.05 was considered to be statistically significant.

## 3. Results and Discussion

### 3.1. The Characterization of GR-Containing MSCS Scaffolds

**Figure 1:**
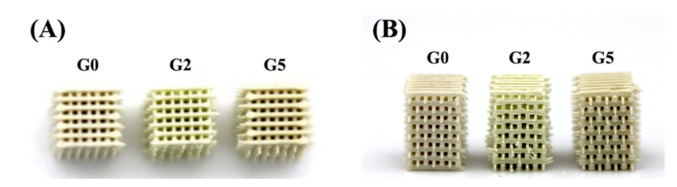
(**A**) The top view and (**B**) side view photographs of the GR-contained MSCS scaffolds.

According to our previous reports, MSCS had a high potential for bone regeneration as it was able to provide a favorable and suitable microenvironment for cells. In this study, we hypothesized that modification with GR could further enhance the osteogenic capabilities of MSCS. GR is a common compound used in traditional medicine in treating systemic bone diseases. As seen from Figure 1, it was possible to fabricate scaffolds with interconnected pores and well-defined struts. Inter-connectivity and pores are important features for bone regenerative scaffolds as pore sizes of between 300–550 μm have been reported to enhance cellular adhesion and subsequent bone regeneration. In a previous study, 3D scaffolds with a pore size between 200 to 500 µm were recommended for bone regeneration because of the high initial cell attachment, reasonable migration, and inhibition of cell aggregation [9]. The struts were neatly stacked on top of one another and the distance between each strut was similar to our original designs. The scaffolds were designed to have a dimension of 6.5 mm × 6.5 mm × 6.5 mm with 500 μm struts. In this study, the scaffolds were labeled G0, G2, and G5 according to the different concentrations of GR-contained MSCS scaffolds. It could be noted that as the concentration of GR increases, the color of the scaffold gradually became darker from the original whitish shade to creamy-white shades. Furthermore, it could be noted that the printing process was smooth and each scaffold had high reproducibility. This indicated the GR-contained scaffold with different designs and structures can be manufactured via the 3D-printing process as needed in the future.

**Figure 2:**
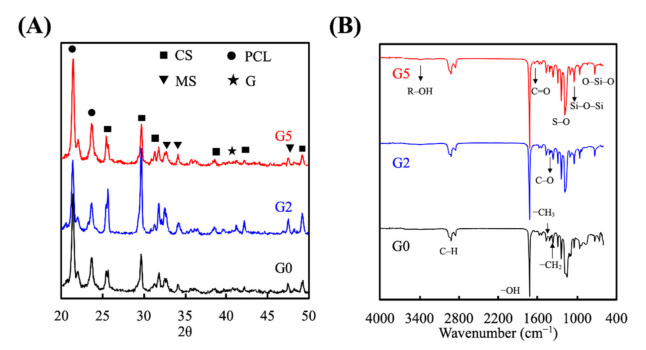
(**A**) X-ray diffractometry and (**B**) Fourier transform infrared spectroscopy results for the GR-contained MSCS scaffolds.

XRD was used to study the interphase patterns, which is important to determine if the GR was successfully mixed into our MSCS powders. The GR composite scaffolds at various conditions under standard spectrum were demonstrated in Figure 2. G0 presented a typical characteristic crystal peak which indicates the presence of a self-crystal structure. The spectra of CS-characteristic peaks were found at 2θ = 25.4°, 29°, 31.8°, 38.6°, and 49.2° [29]. MS-characteristic peaks were detected at 2θ = 31.7°, 32.8°, 47.6° [2]. G2 and G5 had similar GR peaks at 2θ between 22° to 23° (Figure 2A). It can be demonstrated that the inclusion of GR in the scaffold will not affect the main phase structure of MSCS. Compared to G0, G5 had a high crystallinity sharp peak showing at 2θ = 41°, which can represent the fact that the scaffold contains ginsenoside [30]. The functional groups of the synthesized GR-loaded MSCS were analyzed using FTIR spectrophotometry as shown in Figure 2B. The infrared spectra were recorded in the wavelength range from 500 cm^−1^–4000 cm^−1^. The characteristic absorption band in the 1047 cm^−1^ region of the FTIR spectrum was related to the organic components (Si-O-Si) of the MSCS concentration [31]. With the addition of GR, there was a significant increase in FTIR spectra at the 1047 cm^−1^ and 1087 cm^−1^ region [25]. The strength of the characteristic peak increased with the increase of the GR content which suggested that GR transformed the S, O, and Si groups’ structure. Moreover, the spectrum of GR depicts characteristic absorption bands at 3350 cm^−1^ due to the –OH groups.

**Figure 3:**
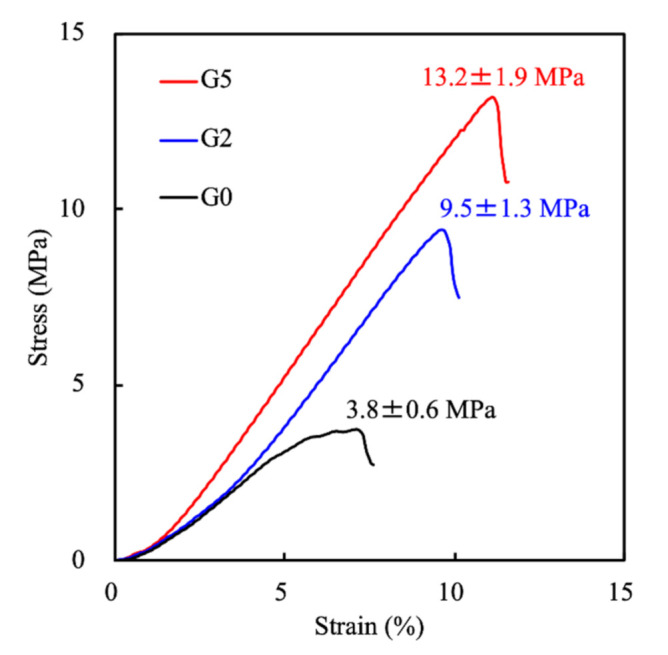
Stress-strain curve of G0, G2, and G5 scaffolds. Data presented as mean ± SEM, n = 6 for each group.

A suitable bone regeneration scaffold needs to have enough mechanical stress to bear the loading of the surrounding bone tissue environment. An EZ Test machine was used to investigate whether the GR extraction could have an additive effect to enhance stress and strain levels. The mechanical properties presented in stress-strain profiles were as shown in Figure 3. As seen, all scaffolds were able to tolerate a sharp increase in compressive stress. The compressive strength of G5, G2, and the G0 scaffold was 13.2, 9.5, and 3.8 MPa, respectively, with G5 having a 3.4-fold increase in compressive strength as compared to the control G0 group. On the other hand, the G2 scaffold had a 2.6-fold increase in compressive strength as compared to the control G0 group, thus indicating that the presence of GR enhanced the mechanical properties of MSCS scaffolds. These results further displayed that those mechanical properties of MSCS scaffolds were directly related to increasing GR concentrations; thus, the addition of GR makes scaffolds more suitable for clinical applications [32]. In this study, we have fabricated a GR-contained MSCS scaffold with proper mechanical behavior. It was also reported that scaffolds with suitable mechanical properties could enhance tissue filling during bone regeneration [33]. GR-contained MSCS scaffold indicated had good mechanical properties that would help assist and resemble the formation rate of new bone formation [34,35].

### 3.2. Immersion Behaviors

**Figure 4:**
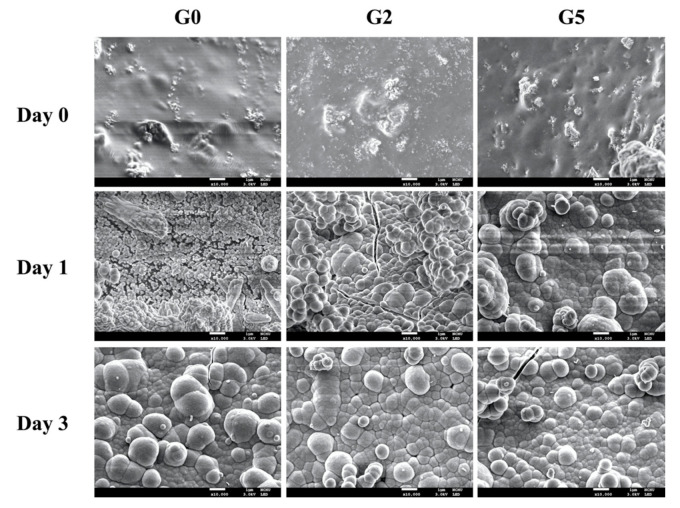
Surface microstructure of the G0, G2, and G5 scaffolds before and after immersion in SBF for 1 and 3 days. The scale bar is 1 μm.

The surface morphology of G0, G2, and G5 scaffolds was observed using SEM and shown in Figure 4. As seen, there were significant changes before and after immersion in SBF as compared to days 0 to 3. G0 had a rather smooth and flat surface on day 0 whilst the GR group (G2, G5) had rougher surfaces hypothesized to be due to the addition of GR. After 1 day of immersion, all groups had hydroxyapatite formation on the surfaces of the scaffolds. However, it could be noted that the hydroxyapatite aggregates were larger on the G5 group as compared to G2 and G0. In addition, the GR groups’ (G2 and G5) surfaces showed the rapid formation of hydroxyapatite aggregates which were uniformly distributed throughout the scaffold. After 3 days of immersion, hydroxyapatite aggregates on the GR groups were seen to be larger and more tightly arranged, and better attached to the scaffolds as compared to G0. Hydroxyapatite formation is commonly used as an indicator for subsequent bone tissue regeneration capability. Several studies indicated the essential requirement for a scaffold to bond to living bone is the formation of this hydroxyapatite layer on the surfaces of interest [36]. In addition, this nano-layer is similar to bone tissue in terms of composite and structure; therefore, primary cells had preferred such an interface for differentiation and subsequent bone regeneration [37]. Therefore, we hypothesized the precipitated hydroxyapatite further increases bonding between the scaffold and native tissue, thus leading to enhanced tissue regeneration.

**Figure 5:**
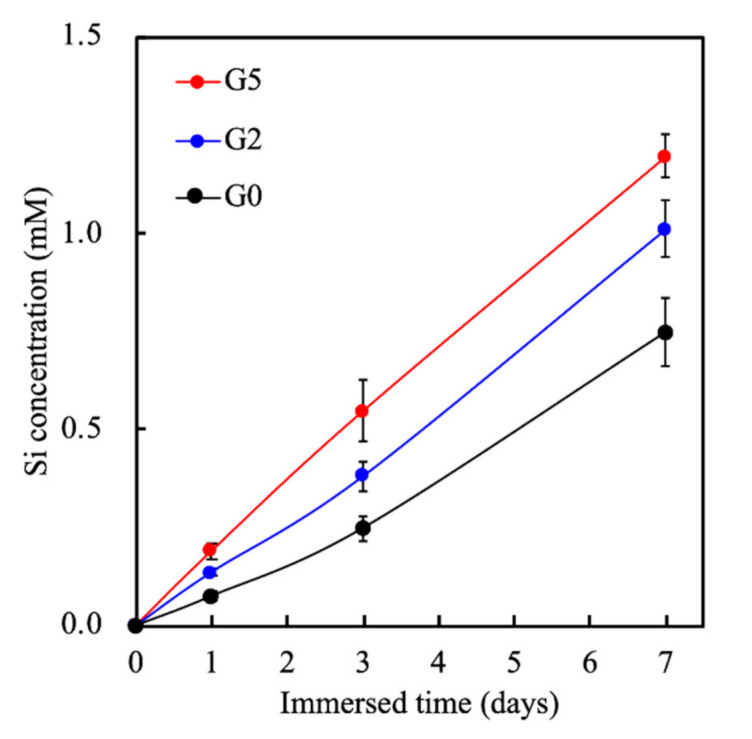
The Si ion concentrations in SBF after soaking for different durations. Data presented as mean ± SEM, n = 6 for each group.

The accumulative release of Si ions into the surrounding medium was analyzed and shown in Figure 5. As seen, sustained release of Si ions was noted for all scaffolds, with G5 having the highest Si concentration at all time points. At the end of 7 days of immersion, the release of 1.2 mM, 0.95 mM, and 0.65 mM of Si ions was noted from G5, G2, and G0, respectively. In fact, the MSCS scaffold is already a material that can regulate cell behaviors, mainly because it spontaneously releases Si ions to promote the proliferation and differentiation of various primary cells [38,39]. Moreover, Si ions were known to enhance collagen secretion and angiogenesis in the mid-stages of bone regeneration [17].

### 3.3. Cell Proliferation of hDPSCs Cultured on GR-Contained MSCS Scaffolds

**Figure 6:**
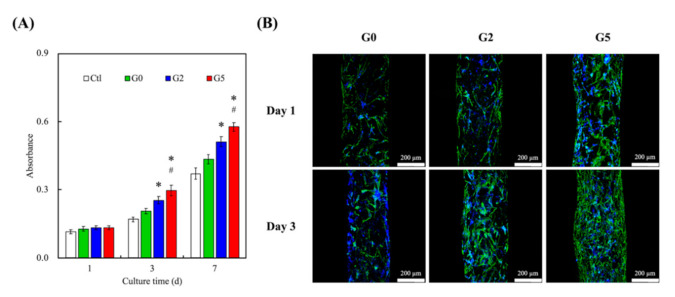
(**A**) Proliferation rate and (**B**) F-actin staining of hDPSCs cultured on the GR-contained MSCS scaffolds for different days. * indicates a significant difference (*p* < 0.05) from G0. # indicates a significant difference (*p* < 0.05) from G2. Data presented as mean ± SEM, n = 6 for each group.

The adhesion, viability, and proliferation of hDPSCs were evaluated and shown in Figure 6A. After day 1, the results of the PrestoBlue^®^ assay revealed that all scaffolds had similar proliferation rates and viability. However, a significant difference (*p* < 0.05) for hDPSCs’ proliferation rates and viability was noted on G2 and G5 scaffolds from day 3 onwards. In addition, a significant difference (*p* < 0.05) for hDPSCs’ proliferation rates and viability were noted on G5 scaffolds from day 3 onwards as compared to G2. These results clearly indicated that GR was able to enhance proliferation rates and viability and that increasing concentrations of GR were able to bring about additional increased effects. It was hypothesized that GR was able to enhance such cellular activities via activation of BMP2/SMAD receptors as reported by others [26]. Confocal microscopy for immunofluorescent was done and results were as shown in Figure 6B. F-actin was stained green whilst the nucleus of cells was stained with DAPI blue. As seen in Figure 6B, G5 had the most DAPI cell nucleus stain at day 3 as compared to the other groups. In addition, it could be noted that on day 1, the F-actin of cells at GR5 was well-spread and covered a larger area as compared to the others. This showed that cells were well-adhered to the surfaces of the scaffolds, thus indicating that the addition of GR made adhesion surfaces more favorable for cellular adhesion and attachment. Reports were made stating that initial levels of cellular adhesion could be used to predict subsequent cellular activities [40]. Therefore, further studies were required to determine if GR had positive effects on subsequent bone regeneration.

### 3.4. Anti-Inflammation

**Figure 7:**
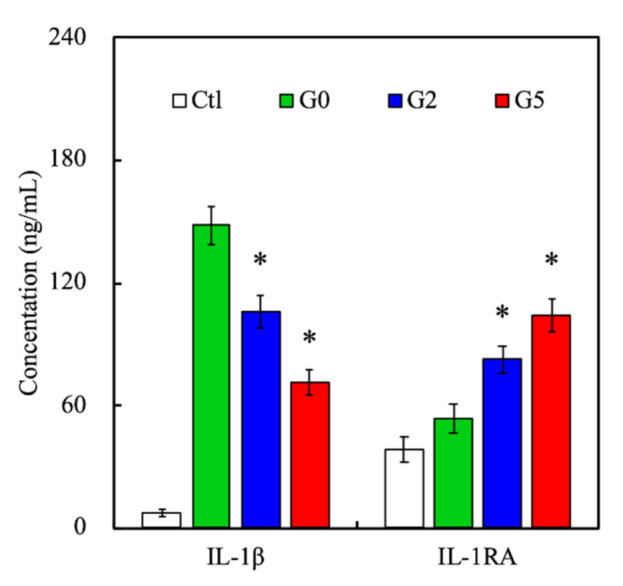
IL-1β and IL-1RA expressions of hDPSCs cultured on the GR-contained MSCS scaffolds for 24 h. * indicates a significant difference (*p* < 0.05) from G0. Data presented as mean ± SEM, n = 6 for each group.

In this experiment, we selected general markers IL-1β and IL-1RA which were useful to identify the inflammatory response [41]. IL-1RA is a well-known antagonist to IL-1 receptors which is functional to anti-inflammation [42]. The CS scaffold has been reported to increase inflammation markers’ up-regulation while seeded on RAW 264.7 that suppressed the cells physiological performance [43]. It can be speculated that the -OH functional groups of CS combined with alkaline media stimulates hDPSCs’ autocrine functions to secrete inflammation cytokines to recruit other inflammatory factors. Therefore, tinflammatory cytokine expression was assessed by ELISA assay as shown in Figure 7. After hDPSCs were cultured on the scaffold for 24 h, the GR-contained MSCS scaffolds could reduce inflammation level, obviously while the GR concentration increased. The control group was absent cement of the cell culture. Scaffolds were assessed according to the culture media of the scaffold to analyze cell-transported messages while culturing. As can be seen, the inflammatory cytokine IL-1β was raised more quickly in the G0 scaffold than control, G2, and G5 scaffolds. However, the expression of G5 was the lowest, which implied cells adapt to the environment of the G0 scaffold only better than the control group while the addition of GR scaffold had an anti-inflammatory effect that can effectively reduce IL-1β expression [44]. Continuously, we investigated another anti-inflammation cytokine IL-1RA to verify that GR could fulfill an anti-inflammatory role in this study. The characteristic of IL-1RA displayed excellent expression in G5 and G2 scaffolds than other groups after being cultured 24 h. The G5 scaffold was able to induce higher secretions of IL-1RA in 24 h, suggesting that GR could still retain the characteristics of drug extracts after mixing with MSCS. As mentioned, GR had a high potential for regulating hDPSCs’ behavior and bone remodeling for dental implants [28]. Our results showed that GR could boost hDPSC proliferation and attachment via F-actin staining because of surface geometry, mechanical properties, and good control-released nutrients. It was important to note that MSCS and GR mixing had an additive effect on cell growth in vitro. These findings indicated GR was nontoxic to cells even after 24 h of culture and had significant differences in anti-inflammation. Compared with other in vitro results, the GR series scaffold was similarly found to provide good biocompatibility and bioactivity environment as well as a candidate to verify mineralization efficiency during the in vivo experiment.

### 3.5. Bone Regeneration-Related Protein Expression

**Figure 8:**
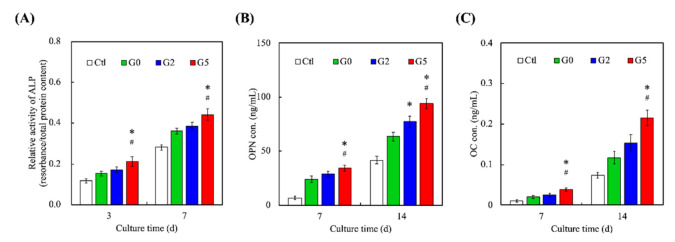
Osteogenic-related differentiation markers of (**A**) ALP activity (**B**) OPN, and (**C**) OC expression of hDPSCs cultured on different scaffolds for different time-points. * indicates a significant difference (*p* < 0.05) from G0. # indicates a significant difference (*p* < 0.05) from G2. Data presented as mean ± SEM, n = 6 for each group.

**Figure 9:**
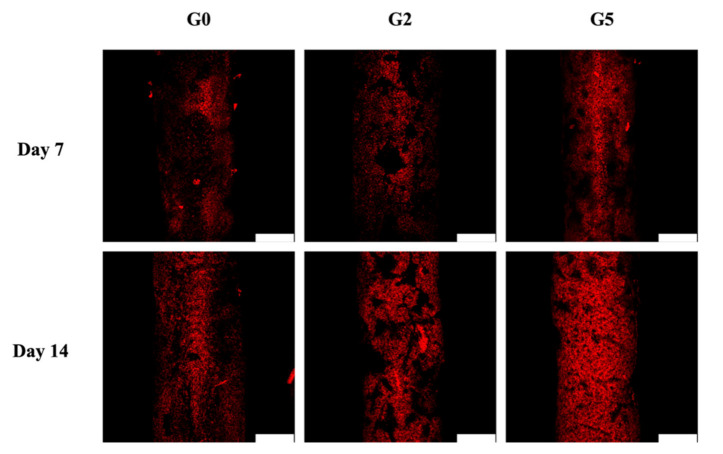
Alizarin Red S staining of hDPSCs calcium mineral deposits cultured on the GR-contained MSCS scaffolds for 7 and 14 days. The scale bar is 200 μm.

The levels of osteogenesis-related markers such as ALP, OPN, and OC were assessed and results were as shown in Figure 8. For ALP, it could be seen that G5 had significantly higher expressions on both days 3 and 7 as compared to Ctl, G2, and G0. Similar trends were seen for both OPN and OC expressions. ALP is an important regulator of bone mineralization and is involved in hydrolyzing inorganic pyrophosphate which is known as a strong inhibitor of bone regeneration. In addition, ALP is also known to provide inorganic phosphates for bone mineralization. On the other hand, OPN is reported to have multiple functions in bone regeneration, of which the presence of OPN strongly leads to enhanced cell adhesion, signaling, and mineralization. In addition, OPN is known to integrate existing bones with newly regenerated bone tissues [45]. Together with OC, these results showed that GR could enhance bone regeneration [46]. These data indicated the osteogenic differentiation of hDPSCs by the expression of ALP, OPN, and OC that are considered important osteogenic-related biomarkers of osteogenic cells and further present a functional proof of bone tissue regeneration. A similar trend was seen by Alizarin Red S staining which was the indicator of Ca mineral deposition associated with later-stage bone regeneration [47]. As seen, levels of bone mineralization for all groups increased as the number of days increased (Figure 9). Even so, it could be seen that G5 had the most matrix deposition and calcium mineral nodules as compared to the other groups on both 7 and 14 days. These findings were confirmed by the amplified expression levels of osteogenic-related protein markers. The results illustrated that GR-contained MSCS scaffolds provide an excellent microenvironment for bone mineralization and subsequent bone regeneration. Therefore, it could be concluded that G5 had more osteoinductive effects as compared to G2 and G0.

### 3.6. Immunohistochemistry in Critical-Size Bone Defect in Rabbit Model

**Figure 10:**
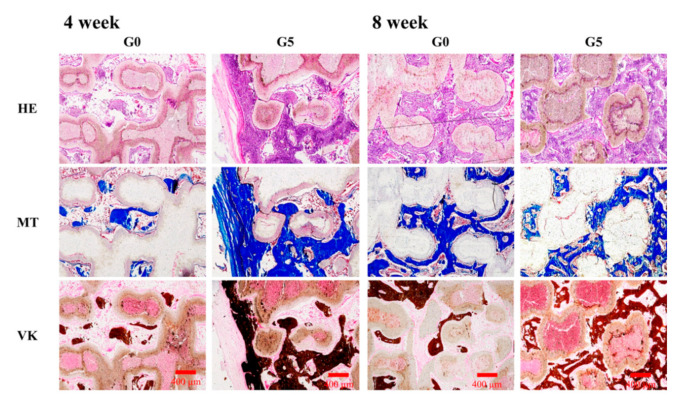
Hematoxylin-eosin (HE), Masson’s trichrome (MT) and von Kossa (VK) staining evaluating new bone regeneration quality of G0 and G5 scaffolds in a critical-sized bone defect in vivo at 4 and 8 weeks of implantation. The scale bar is 400 μm.

The overarching aim of developing bone regeneration biomaterials is to repair broken bone tissues, and the act of bone substitute scaffolds presently depends on the ability to perform osteogenic functions in vivo [3]. The various scaffolds were implanted into bone defect animal models and histological assays of the scaffolds were as shown in Figure 10. As seen, the GR-contained MSCS scaffolds had markedly increased collagen formation, mineralization of bone defect area, and greater proportions of calcified hard tissue as compared to the others. We further examined the GR scaffold via H&E, Masson’s trichrome (MT), and von Kossa staining (VK) and found it could promote bone regeneration and bone formation effectively in vivo [48]. Consistent with the in vitro results, H&E staining indicated that the hard tissue morphology presented more tibia integrity in the G5 scaffold groups than the G0 scaffold [49]. MT and VK staining also showed that collagen and calcification were increased in the G5 scaffold after 4 and 8 weeks of implantation. The defects in the G0 group are still visible. Moreover, The G5 scaffolds were progressively degraded by the newly formed bone tissue during regeneration [50]. Therefore, the GR-contained MSCS scaffold was shown to be beneficial for cellular adhesion and attachment, which led to enhanced bone regeneration of bone defects.

## 4. Conclusions

In this study, GR extracts were blended with MSCS and successfully printed into 3D scaffolds. The 3D-printed scaffolds had homogeneous pore morphology, uniform porous structures, and was able to induce hydroxyapatite formation rapidly after immersion in SBF solution. It was worth noting that the compressive strength of the scaffolds increased significantly with GR modification. G5 scaffolds had a mechanical strength that was 3.6-fold higher than the control group G0. Cellular behavior studies showed that GR promoted proliferation responses of hDPSCs as well as higher mineralization rates when compared to the control group. Histological results further confirmed the in vivo bone regeneration capability of GR. These results strongly indicated that 3D-printed GR scaffolds have great potential in bone tissue engineering. We showed that it is possible to think out of the box to combine current biomaterials and technology with existing treatment methods to enhance and obtain maximal benefits of bone regeneration. 

## Data Availability

Data are available in a publicly accessible repository.
